# Job Satisfaction among Swedish Pharmacists

**DOI:** 10.3390/pharmacy8030127

**Published:** 2020-07-24

**Authors:** Sofia Mattsson, Maria Gustafsson

**Affiliations:** Department of Integrative Medical Biology, Umeå University, SE-901 87 Umeå, Sweden; maria.gustafsson@umu.se

**Keywords:** job satisfaction, pharmacy graduate, continuous professional development

## Abstract

Understanding the role of pharmacists’ job satisfaction is important because a lack of job satisfaction might have negative impacts on patient care and safety. The aim of this cross-sectional study was to explore and compare job satisfaction among pharmacists graduating from the pharmacy programs at Umeå University, Sweden. Data concerning job satisfaction and associated factors were collected using an alumni survey conducted among pharmacists graduating between 2015 and 2018. Ethical committee approval is not required for this type of study in Sweden. A majority (92.6%) of the pharmacy graduates were female. A majority of the graduates (91.4%) were satisfied with their job most of the time or all of the time, which was similar to a previous investigation among pharmacists graduating between 2006 and 2014. High access to continuous professional development (CPD) was associated with higher job satisfaction (odds ratio (OR): 18.717 (95% confidence interval (CI): 1.685–207.871)). In total, 65.6% considered access to CPD to be high (i.e., satisfactory to very good). Variables like gender, age, employee category, workplace, years since graduation, and income did not affect job satisfaction. Knowledge regarding job satisfaction will enable employers to respond to employees’ needs, decrease turnover, and improve the work environment.

## 1. Introduction

In Sweden, as in many other countries, the roles of pharmacists have changed over the years. From primarily dispensing medications, pharmacists have become more clinically involved in patient care in different ways in the healthcare system [1]. For example, clinical pharmacists as part of a ward team have become more common in hospitals and primary care, and patient care services and different business models have also been developed in community pharmacies. Some of these changes in job assignments might be due to the re-regulation of the Swedish pharmacy market that took place in 2009. This re-regulation implied a transition from a state-owned pharmacy monopoly to an open pharmacy market, including both community and hospital pharmacies [1,2,3,4]. Taken together, these changes might have affected pharmacists’ job satisfaction. This is important because performance, motivation, and productivity are factors that are positively linked to job satisfaction, while lack of job satisfaction might affect patient care and safety negatively and increase job turnover [5,6,7,8,9]. 

Several studies have addressed pharmacists’ job satisfaction with different results. At an international level, a study performed in Saudi Arabia found that most of the pharmacists in different healthcare settings were satisfied with their current job (39% satisfied and 25% slightly satisfied) [6]. A recent study in Malaysia found that job satisfaction among community pharmacists was high (77% were satisfied with their jobs) [10], whereas another study in Malaysia among pharmacists in the public sector reported that only 52% were satisfied with their current job [11]. Further, in Northern Ireland, a little more than half of the community and hospital pharmacists reported that they were satisfied with their current job “most of the time”, and in that study, job satisfaction was linked to stress levels [12]. Many factors might contribute to pharmacists’ job satisfaction and dissatisfaction, according to previous research. For example, lack of time for interaction and lack of recognition can affect job satisfaction negatively [13]. The setting seems to be important, and one study performed in Great Britain found that community pharmacists were less satisfied compared to pharmacists working in other sectors [14]. Further, continuing professional development (CPD) has been found to be of importance for job satisfaction [15], as well as the female gender [16]. Age, income, and part-time work are other factors associated with job satisfaction [11,16,17,18].

Umeå University offers three pharmacy programs—a three-year Bachelor of Science in Pharmacy program, a five-year Master of Science in Pharmacy program, and a two-year Master of Science in Pharmaceutical Science program. The programs were started in 2003, 2012, and 2010, respectively. Graduates with a Bachelor of Science in Pharmacy degree can apply to the two-year master’s program in order to obtain a master’s degree. In addition, there are two different professional degrees in Sweden prescriptionists (with a bachelor’s degree) and pharmacists (with a master’s degree). The term pharmacist will be used throughout the paper, including both professional degrees. All three pharmacy programs are web-based, and the online material consists of recorded video lectures, assignments, and animations and is available through a virtual learning environment. Meetings between students and teachers occur online approximately once a week. In addition, the students are gathered on campus 2–4 times each semester for lab work, oral presentations, and role play.

A questionnaire was previously sent out to those who had graduated from pharmacy programs at Umeå University, Sweden, between 2006 and 2014 [19]. That study found that most graduates (91%) were satisfied with their jobs. The study also found that the factors of access to CPD and the perception that the knowledge and skills acquired during university education are useful in the current job were significantly associated with job satisfaction. Because of the aforementioned changes in pharmacists’ roles and assignments, it would be interesting to investigate the present level of job satisfaction and if there have been any changes over time. The aim of the present study was, therefore, to explore job satisfaction among pharmacists graduating from the pharmacy programs at Umeå University, Sweden, between 2015 and 2018, and to compare this with those graduating between 2006 and 2014. 

## 2. Materials and Methods 

### 2.1. Setting

This cross-sectional study was conducted among pharmacists who graduated from Umeå University in Sweden between 2015 and 2018. There are four universities offering pharmacy education in Sweden, and all of them are public universities. The curricula of all pharmacy programs are similar and include courses in chemistry, biomedical science, physiology, pathology, toxicology, pharmacology, pharmacokinetics, pharmacotherapy, pharmaceutics, and pharmacy practice. The Swedish Higher Education Authority is the centralized accrediting body, responsible for the evaluation of the quality of higher education and research. There are about 350–400 pharmacists graduating in Sweden annually, of which about 60 graduates are accounted for by Umeå University [20]. In total, there are about 10,000 registered pharmacists in Sweden [21].

### 2.2. Survey

A questionnaire was sent out to those who had graduated from the Bachelor of Science in Pharmacy, Master of Science in Pharmacy, and Master of Science in Pharmaceutical Science programs at Umeå University. The total number of graduates was 222, and 16 graduates had earned both a bachelor’s and a master’s degree. The paper questionnaire was sent out in February 2019 to the graduates by post to the address registered in the university’s administrative register. Addresses in the university´s administrative register are continuously updated against the Swedish Tax Agency, which manages the civil registration of private individuals in Sweden. Together with the questionnaire, a postage-paid envelope was included. The graduates were asked to return the questionnaire by 15 March 2019. Sixteen graduates did not live in Sweden and consequently did not have an address in the university´s administrative register, and to those, an e-mail was sent with an invitation to participate in the survey. Two of those accepted to participate in the survey, and the paper questionnaire was sent by post. No reminders were sent out. Data were collected during February and March 2019. Ninety-four graduates completed the survey, giving a response rate of 42%.

In 2015, a similar survey was conducted with graduates graduating between 2006 and 2014, and the present questionnaire was developed on the basis of the previous questionnaire [19]. The questionnaire asked the graduates about their current job and job assignments, their job satisfaction, and their education. Job satisfaction was evaluated using a five-item validated version of the survey from McCann et al. [12]. The questions were translated into Swedish and then back-translated into English. These questions regarding job satisfaction were also used in the alumni survey from 2015 [19]. 

### 2.3. Data Analysis

Data from the present survey in 2019 were compared with data from the survey in 2015, and descriptive statistics were used to summarize the data. Results from the survey in 2015 were published previously [19]. The basis for comparing the present study with the previous one was to enable job satisfaction to be studied over time. The investigation in 2015 was the first to study job satisfaction among pharmacy graduates at Umeå University, and this group may, therefore, be considered as a baseline. Job satisfaction was measured with the question, “All things considered, how often are you satisfied with your job?”, and the answers were dichotomized into “not satisfied” (those who responded “never or rarely” and “sometimes satisfied”) and “satisfied” (those who responded “satisfied most of the time” and “satisfied all of the time”). The answers to the questions “how often do you think the idea of spending the remainder of your working life in a job like your current one is depressing”, “how often do you leave work with a ‘bad’ feeling”, and “how often do you get so wrapped up in your work that you lose track of time” were all dichotomized into “never/sometimes” (those who responded “never or rarely” and “sometimes”) and “most of the time/all of the time” (those who responded “most of the time” and “all of the time”). The answers to the question about access to CPD were dichotomized into “limited” (“limited” and “very limited”) and “good” (“satisfactory”, “good”, and “very good”). The answers to the question of whether “the knowledge and skills you acquired during your training are useful in your current job” were dichotomized into “disagree” (1–3 points) and “agree” (4–5 points). The answers between the groups were compared using the chi-squared test. 

Data from the paper questionnaires were manually compiled using Excel. Simple logistic regression analyses were conducted to investigate the association between job satisfaction and the factors of age, gender, employee category, current employment, income, years since graduation, access to CPD, and if the knowledge and skills they acquired during their training are useful in their current job. Most of these factors were found to be associated with job satisfaction in previous research and were, therefore, of interest in the present study [11,14,15,16,17,18]. A multiple logistic regression analysis was conducted, including significant variables from the simple models, and these also included age and gender. Results are presented as odds ratios (ORs) with 95% confidence intervals (CIs). The significance level for all statistical tests was set at 5%. All analyses were conducted using Statistical Package for the Social Sciences (SPSS) for Windows version 26 (IBM, Armonk, New York, NY, USA).

### 2.4. Ethics

This type of study does not require ethical committee approval in Sweden. All respondents were given information about the aim of the study and that the data would be treated as strictly confidential and that all answers would be anonymous.

## 3. Results

### 3.1. Characteristics of the Graduates 

A majority (92.6%) of the pharmacists graduating were female, and two-thirds graduated from the three-year bachelor program (Table 1). The average age ranged between 33.4 and 37.0 years, the oldest being the graduates with a master’s degree. Furthermore, most of the graduates were born and currently live in Sweden. Over 80% of the graduates with a bachelor’s degree worked in community pharmacy, while among the graduates with a master’s degree, a greater diversity was seen regarding the workplace. A majority of the graduates had permanent employment and had gotten their first job before graduating. 

### 3.2. Job Satisfaction

Job satisfaction was evaluated using a validated five-item scale (Table 2). The results showed that a majority of the graduates (91.4%) were satisfied with their job most of the time or all of the time. When comparing the current results with the previous study, the overall job satisfaction was similar (91.4% versus 91.2%, *p* = 0.965) [19]. However, a greater proportion in the present study thought that spending the remainder of their working life in a job like their current one was depressing most of the time or all of the time (13.0% versus 3.8%, *p* = 0.003) [19]. The other questions regarding job satisfaction showed similar results between the present study and the previous study (*p* > 0.05) [19]. When comparing job satisfaction among graduates with a bachelor’s and a master’s degree, no differences were observed (*p* > 0.05). 

**Figure 1:**
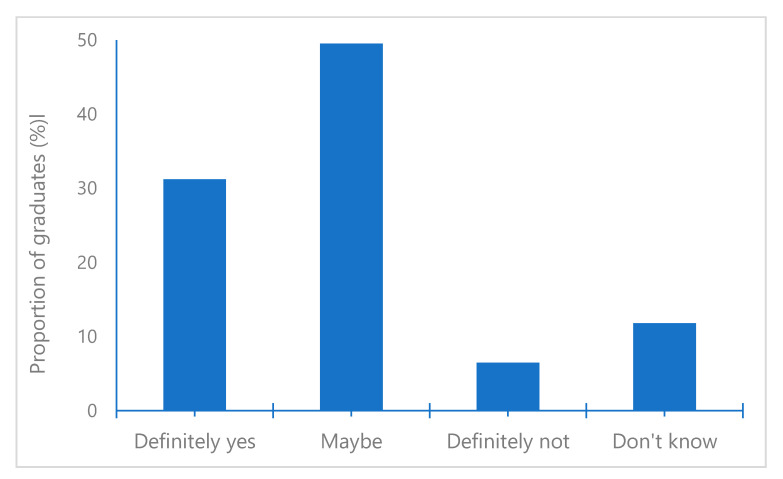
Responses to the question of whether the graduates would start the same career if they were to choose again (graduation 2015–2018).

To the question of whether the graduates would start the same career if they were to choose again, 80.7% answered “definitely yes” or “maybe” (Figure 1). A majority in both the present study and the previous study responded maybe or definitely yes to the question (80.7% versus 86.7%, *p* = 0.172) [19].

**Figure 2:**
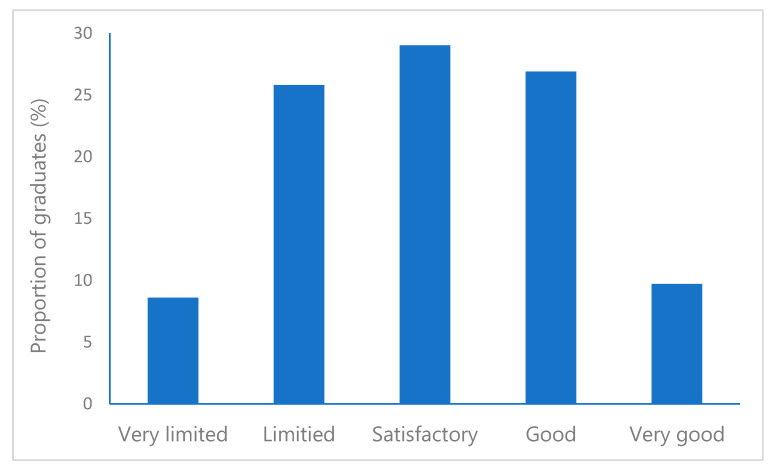
Responses to the question about their possibilities for continuous professional development (CPD) (graduation 2015–2018).

The graduates were also asked a question about their possibilities for CPD, and a majority of the respondents considered access to CPD as high (i.e., satisfactory to very good) (Figure 2). In total, 65.6% considered access to CPD as high in the present study compared with 60.8% in the previous study (*p* = 0.428) [19].

To the question of whether the knowledge and skills acquired during their education are useful in their present job, 82.6% agreed or strongly agreed, which was similar to the corresponding proportion of 87.6% in the previous study (*p* = 0.244) [19].

A regression analysis was conducted to investigate associations between job satisfaction and different factors (Table 3). The simple regression analysis showed that female gender (OR: 6.750 (95% CI: 1.021–44.641)) and high access to CPD (OR: 16.800 (95% CI: 1.964–143.737)) were associated with higher job satisfaction. Furthermore, considering that the knowledge and skills acquired during their education are useful in their present job was also associated with higher job satisfaction (OR: 6.000 (95% CI: 1.391–27.287)), but no association between job satisfaction and other factors was observed. In the multiple regression analysis, only high access to CPD was associated with higher job satisfaction (OR: 18.717 (95% CI: 1.685–207.871)). 

## 4. Discussion

This study explored job satisfaction among pharmacy graduates and also compared job satisfaction over time. The characteristics of the graduates in the present study were similar to the previous study [19]. A majority of the graduates were female, were born and lived in Sweden, and worked in community pharmacy. The median age of graduates in Sweden is 26.9 years (2018/2019) [22]. Compared to this and also to pharmacy graduates at other universities in Sweden, graduates from Umeå University are older, which is likely due to the web-based format of education [23]. A majority of the graduating pharmacists were female, and this is consistent with the pharmacy student population and the workforce of pharmacists in Sweden as well as in other countries [24,25,26,27,28]. Besides, there were similarities in age and gender between the alumni survey and the university records, showing that the sample was representative of the graduates.

The overall job satisfaction was high. No differences in job satisfaction were observed between graduates with a bachelor’s and a master’s degree. Among graduates with a master’s degree, a greater diversity was seen regarding the workplace, which is expected since a master’s degree offers greater possibilities to work in other workplaces besides community pharmacy. A majority of the graduates worked in community pharmacy, which has undergone substantial changes during the last decade, partly as a result of the re-regulation of the pharmacy market in 2009. The number of community pharmacies has increased [1], the opening hours have been extended, and the demand for prescriptionists and pharmacists has increased. The results showed that most graduates obtained their first job even before graduation, which confirms the high demand for prescriptionists and pharmacists in Sweden. Furthermore, the roles, as well as job assignments of pharmacists in community and hospital pharmacy, have changed. A recent investigation regarding the work environment was performed among Swedish pharmacists working in community pharmacies, and the pharmacists reported high workloads and feelings of stress [29]. Previous studies have shown an association between workload and job satisfaction and stress among pharmacists, i.e., a high workload and stress negatively affect job satisfaction [30,31,32,33,34,35]. The changes in the pharmacy market over the last decade might have the potential to negatively affect job satisfaction, but this was not observed. 

Compared to other studies investigating job satisfaction both in Sweden and in other countries [11,29,32,35,36,37,38], the present study, as well as the previous investigation in 2015 [19], showed that job satisfaction among the graduates was higher. Furthermore, a majority of graduates answered maybe or definitely yes to the question of whether they would start the same career if they were to choose again, thus indicating that most of the graduates were satisfied with their choice of career. Comparisons with other countries must be made with caution due to different contexts, but studies of job satisfaction in Sweden are limited. The higher job satisfaction among the graduates might at least partly be explained by the web-based format of the pharmacy programs, which attracts certain types of students, i.e., highly motivated students with a great interest in pursuing a career within pharmacy [19]. However, because a higher proportion in the present study, compared to the previous study [19], thought that spending the remainder of their working life in a job like their current one was depressing most of the time or all of the time, this might indicate that job satisfaction is decreasing, and further investigations are needed in order to confirm this.

No effects of gender on job satisfaction were observed in this study. However, other studies have shown that gender can affect job satisfaction and that women in general report higher levels of job satisfaction [16,39,40]. In addition, older age has also been shown to be associated with higher job satisfaction [11,14,19]. However, in the present study, age was not shown to affect job satisfaction, which could be due to the sample size. The result regarding age was in agreement with a recent study conducted in Malaysia, where levels of job satisfaction were shown not to be related to age [10]. A U-shaped age effect on job satisfaction was also reported earlier, i.e., younger and older workers showed higher job satisfaction than middle-aged workers [16]. Thus, the effect of age on job satisfaction appears to be complex.

A majority of the graduates agreed or strongly agreed that the knowledge and skills acquired during their education are useful in their present job. However, in contrast to the previous study [19], this was not associated with higher job satisfaction, which might be due to the sample size. The results from the present survey showed that a majority of the respondents considered that their access to CPD was high (satisfactory to very good). High access to CPD was also associated with high job satisfaction, which was in line with previous research [15,19]. However, approximately one-third of the respondents had limited or very limited access to CPD, indicating that there is still room for improvement when it comes to access to CPD in Sweden [19].

Other investigated variables like employee category, workplace, years since graduation, and income did not affect job satisfaction in this study. The workplace was previously found to affect job satisfaction [10,14,18]. One study found that community pharmacists were less satisfied compared to pharmacists working in other sectors [14]. Another study found that job satisfaction was higher among pharmacists at chain pharmacies compared to pharmacists at independent pharmacies [10], but the opposite was also found [18]. Regarding years since graduation, this variable appeared to both increase [11], decrease [19], or be independent of job satisfaction [10] in previous research. Income was also found to both increase [18] or be independent [19] of job satisfaction. The variation in results regarding variables that affect job satisfaction is probably an effect of different settings and circumstances in different countries. Some of the investigated variables in this study resulted in rather large confidence intervals in the regression analysis, which might be due to the relatively small sample size or a large variation within groups.

### Strengths and Limitations of the Study

This study has some strengths and limitations. An advantage is that the study enabled job satisfaction to be studied over a relatively long time period, i.e., between 2006 and 2018, when comparing two groups. However, job satisfaction can be regarded as a subjective matter and might vary depending on individual preferences and might also vary over time for one individual. The questions about job satisfaction were asked at a single time point, and this has to be taken into account when interpreting the results. Furthermore, the results might not be representative of the pharmacist workforce in Sweden because all participants had graduated from the same university. It would be interesting to conduct a study, including pharmacy graduates from other universities in Sweden, in order to explore if there are any differences regarding job satisfaction due to regional differences or differences in the educational set up (i.e., campus versus distance-based education). In addition, selection bias cannot be excluded because graduates who are more positive with regards to their profession might have been more likely to answer the survey compared to those who are more negative.

## 5. Conclusions

This study investigated job satisfaction among pharmacists graduating between 2015 and 2018 and showed that the overall job satisfaction was high. Furthermore, when comparing the results with a previous study (pharmacists graduating between 2006 and 2014), the level of job satisfaction was similar. The changes in pharmacists’ roles and assignments occurring over the past years appeared not to have impacted on the level of job satisfaction. The factor of most importance for job satisfaction was access to CPD, and high access to CPD was associated with high job satisfaction. No effects of gender or age on job satisfaction were observed. In addition, variables like employee category, workplace, years since graduation, and income did not affect job satisfaction. High job satisfaction among pharmacists might affect performance, motivation, and productivity positively, while a lack of job satisfaction might affect patient care and safety negatively and increase job turnover. Knowledge regarding job satisfaction will enable employers, managers, and policymakers to respond to employees’ needs, decrease turnover, and improve the work environment. It will also enable educators to work with student recruitment strategies and prepare students for their future professional roles.

## Figures and Tables

**Table 1:** Characteristics of respondents participating in the survey (the graduation year 2015–2018). As a comparison, age and gender were also taken from the university’s administration system.

Characteristic	All Graduates n = 94	Bachelor’s Degree n = 63	Master’s Degree n = 31 *
*Gender (alumni survey) (%)*			
Women	92.6	96.8	83.9
*Gender (university admin. system) (%)*	*(n = 222)*		
Women	89.6	92.0	84.7
*Age (alumni survey)*			
Mean	35.0	34.4	36.3
Standard deviation	8.6	9.2	7.0
*Age (university admin. system)*	*(n = 222)*		
Mean	34.5	33.4	37.0
Standard deviation	8.3	8.3	7.8
*Country of birth (%)*			
Sweden	77.2	82.3	66.7
Outside Sweden	22.8	17.7	33.3
*Country of living (%)*			
Sweden	98.9	100.0	96.8
Outside Sweden	1.1	0	3.2
*Years since graduation*			
Mean	2.7	2.8	2.5
Standard deviation	1.1	1.0	1.4
*Occupation (%)*			
Employed	74.5	69.8	83.9
Student	8.5	11.1	3.2
Parental leave	17.0	19.0	12.9
*Current employment (%)*			
Community pharmacy	76.3	81.0	66.7
Hospital pharmacy	4.3	6.3	0
County council **	6.5	3.2	13.3
Pharmaceutical company	3.2	0	10.0
Other ***	9.7	9.6	10.0
*Employee category (%)*			
Employee	67.7	74.6	53.3
Manager	10.8	11.1	10.0
Specialist	18.3	14.3	26.7
Other	3.2	0	10.0
*Type of employment (%)*			
Permanent	89.2	87.3	93.3
Other	10.8	12.7	6.5
*Time until the first job after graduation (%)*			
Before completing education	86.2	88.9	80.6
Less than six months after	10.7	9.6	12.9
More than six months after	1.1	0	3.2
*Personal monthly income* *(SEK before tax)*			
Less than 25,000	4.3	4.8	3.4
25,001–30,000	27.2	33.3	13.8
30,001–35,000	44.6	47.6	37.9
35,001–40,000	10.9	7.9	17.2
40,001–45,000	9.8	3.2	24.1
45,001 or more	3.3	3.2	3.4

* Thirteen of these graduates had also taken a bachelor’s degree at Umeå University. These graduates were counted as graduates with a master’s degree in the analysis, i.e., the highest degree was considered. ** County councils are responsible for the public health care systems in Sweden. *** Other include drug product manufacturing, dose dispensing pharmacy, university, and municipality.

**Table 2:** Questions about job satisfaction among pharmacy graduates (the graduation year 2015–2018). The proportion of graduates choosing the different options in the survey.

Question	The Proportion of Graduates (%)			
	Never or Rarely	Sometimes	Most of the Time	All of the Time
All things considered, how often are you satisfied with your job?	1.1	7.5	80.6	10.8
How often do you think the idea of spending the remainder of your working life in a job like your current one is depressing?	53.3	33.7	9.8	3.3
How often do you leave work with a “bad” feeling, a feeling that you are doing something you do not enjoy?	68.8	26.9	4.3	0
How often do you get so wrapped up (interested) in the work that you lose track of time?	9.8	51.1	35.9	3.3

**Table 3:** Multivariate logistic regression, including different questions regarding job satisfaction among pharmacists graduating 2015–2018.

	People Satisfied with Their Job	People not Satisfied with Their Job	Simple OR (95 % CI)	Multiple OR (95 % CI)
Cases, n (%)	85	8		
Women, n (%)	81 (95.3)	6 (75.0)	6.750 (1.021–44.641)	6.615 (0.314–139.248)
Age mean ± SD	34.8 ± 8.6	37.9 ± 8.5	0.963 (0.892–1.040)	0.988 (0.901–1.084)
Employee category				
Manager, n (%)	10 (11.8)	0 (0)	-	
Employee, n (%)	75 (88.2)	8 (100.0)	Ref	
Current employment				
Community pharmacy, n (%)	64 (75.3)	7 (87.5)	0.435 (0.051–3.747)	
Other, n (%)	21 (24.7)	1 (12.5)	Ref	
Personal monthly income (SEK before tax)				
>30,000	55 (68.8)	5 (62.5)	1.320 (0.292–5.960)	
<30,000	25 (31.3)	3 (37.5)	Ref	
Years since graduation	2.7 ± 1.1	2.9 ± 1.0	0.856 (0.440–1.665)	
Access to CPD				
Good	60 (70.6)	1 (12.5)	16.800 (1.964–143.737)	18.717 (1.685–207.871)
Limited	25 (29.4)	7 (87.5)	Ref	
The knowledge and skills acquired during education are useful in my current job				
Agree, n (%)	72 (85.7)	4 (50.0)	6.000 (1.391–27.287)	3.787 (0.511–28.053)
Disagree, n (%)	12 (14.3)	4 (50.0)	Ref	

CPD = continuing professional development; SEK = Swedish krona, SD = Standard Deviation, OR = Odds Ratio, CI = Confidence Interval. The multivariate model included significant variables (*p* < 0.05) as independent variables; access to CPD; the knowledge and skills acquired during education are useful in my current job. Gender and age were also included.
